# Selenium and Natural Zeolite Clinoptilolite Supplementation Increases Antioxidative Status and Immune Response in Growing Pigs

**DOI:** 10.3389/fvets.2021.688915

**Published:** 2021-07-30

**Authors:** Tomislav Šperanda, Valentina Pavić, Zdenko Lončarić, Marcela Šperanda, Maja Popović, Vesna Gantner, Mislav Ðidara

**Affiliations:** ^1^Faculty of Agrobiotechnical Science in Osijek, J. J. Strossmayer University of Osijek, Osijek, Croatia; ^2^Department of Biology, J. J. Strossmayer University of Osijek, Osijek, Croatia; ^3^Veterinary Faculty, University of Zagreb, Zagreb, Croatia

**Keywords:** antioxidative status, immune response, natural zeolite clinoptilolite, selenium deposition, selenium supplementation

## Abstract

Selenium (Se), an essential trace element for human and animal health, is covalently incorporated into amino acids, acts as a cofactor for antioxidant enzymes, and is involved in the maintenance of the immune system. The main goal of this investigation was to show the effect of Se supplementation, at levels slightly higher than the recommended values, combined with natural zeolite clinoptilolite on Se deposition in tissues (muscle and liver) and on the immune and antioxidative status of supplemented growing pigs. The experiment was carried out during a 98 d period on 60 pigs. Pigs were fed a standard feed mixture based on corn and soybean and were divided into four groups, according to the level of dietary selenium supplementation as follows: C-0.3 mg/kg DM organic Se, E1-0.5 mg/kg DM sodium selenite, E2-0.5 mg/kg DM organic selenium; E3-0.5 mg/kg DM organic Se+0.2% zeolite. Higher (*P* < 0.05) selenium concentrations were determined in the muscle and liver in growing pigs fed with higher organic Se in combination with zeolite compared to the lower organic Se concentration. Addition of organic Se increased (*P* < 0.05) Se deposition in muscle and liver compared to the equal amount of inorganic Se (E2 vs. E1). Higher organic Se in combination with natural zeolite addition increases (*P* < 0.05) proportion of pigs' cluster of differentiation (CD)45^+^ compared to the same amount of inorganic Se and lower organic Se addition. The proportion of CD45^+^ and CD4^+^ lymphocytes was higher (*P* < 0.05) in E3 group compared to the other groups. Higher (*P* < 0.05) proportion of CD21^+^ lymphocytes were measured in the E2 and E3 groups compared with the other groups. The highest (*P* < 0.01) activity of glutathione peroxidase (GSH-Px) in pig erythrocytes was observed in the E3 group, while higher (*P* < 0.05) activity of glutathione reductase (GR) was in all experimental groups related to the control one. A dietary addition of 0.5 mg/kg DM of organic Se in combination with zeolite (0.2% DM) has increased (*P* < 0.05) Se deposition in liver, muscle, and blood, compared to the dietary addition of 0.3 mg/kg DM of the organic Se.

## Introduction

Selenium (Se) is an essential element for humans and animals but not for plants (1). The bioavailability of Se depends on the plant itself as well as on the concentration of Se in the soil (2, 3). Selenium concentrations in three main soil types of the main agricultural region of Croatia (Osijek-Baranja County), namely haplic gelysol, stagnosol, and luvisol, were reported to be 538, 323, and 314 μg/kg, respectively (4), which means that there is a demand for Se supplementation in animal nutrition (5). As a key component of glutathione peroxidase (GSH-GPx) and other selenoproteins, Se is essential and plays a crucial role in various biological processes, such as the fertilization capacity of spermatozoa (6), semen quality and fertility under heat stress conditions (7), innate and adaptive immune responses (8), metabolic rate (9), and reduced accumulation of heavy metals in tissues (10). Many studies have attempted to produce Se-enriched animal products via feed-based nutritional interventions to increase Se deposition in meat, eggs, and milk (11). Therefore, Se-enriched animal products have drawn extensive attention due to their potential to improve the Se health status of human consumers (12, 13). Supplementation of animal feed with the mineral form of Se, also named inorganic form of Se, has some disadvantages, which are related to an interaction with other minerals, relative high toxicity, and inability to build and maintain Se reserves in the body (8). Furthermore, selenite at high doses has been reported to act as a pro-oxidant. It has been suggested that organic selenium is more effective because it reaches tissues more efficiently and therefore the use of organic Se sources, such as selenomethionine has been shown to be superior to that of inorganic sources (14). Zavodnik et al. (15) have demonstrated a 6-percent birth rate increase concerning the piglets fed by Se yeast supplement, an increase in saw litter weight to 11.1% and the concentration of Se in muscle increased up to 27.8%. In post-weaning piglets, an increase in liver and plasma selenium concentrations subsequent to a supplementation with Se from both organic and inorganic sources. Interestingly, the plasma activity of GSH-Px has been found to decrease with the increase in Se supplementation, but the hepatic activity of GSH-Px increases with increasing Se supplementation, regardless of the type of source (16). According to Rovers (17), Se deposition in muscle tissue is a good indicator of the selenium status of animals. The same author strongly suggests that selenized yeast with higher concentrations of selenomethionine significantly increases deposition of Se in muscle tissue compared with selenized yeast with a lower content of selenomethionine.

Natural zeolite clinoptilolite has been used in veterinary and human medicine as feed ingredient due to its beneficial properties as immunostimulant (18) or as an adjuvant in antibiotics delivery (19). One explanation of beneficial immune effects of silica, silicates, and aluminosilicates could be their action as non-specific superantigen-like immunoglobulins (SAg) capable of activating a large population of T-cells. The activation occurs as a result of a simultaneous interaction between SAg, T cell receptor (TcR) variable region β, and major histocompatibility complex (MHC) class II molecules on the surface of antigen presenting cells (APC) (20). In the weaned piglets which have received 0.5% clinoptilolite for 5 weeks, it was observed that the clinoptilolite was effective as an immunomodulatory agent by promoting the recruitment of circulating and intestinal immune cell subsets, even though it has not improved the growth in the weaned pigs, and generally failed to improve their feed conversion efficiency (21). These findings encouraged us to investigate the combined effect of higher Se concentrations and natural zeolite clinoptilolite on Se deposition in pig tissues (muscle and liver) as well as on the immune and antioxidative status of the supplemented animals.

The hypothesis is based on a knowledge that selenium and zeolite, separately, enhance an immune response and exert an antioxidant effect. It was our assumption that a higher amount of organic Se source (0.5 mg/kg DM) in combination with a natural zeolite clinoptilolite, will result in a better tissue deposition when compared with the same amount of inorganic Se and a lower amount of an organic Se source (0.3 mg/kg DM).

## Materials and Methods

### Animals, Feeding, and Experimental Design

The experiment was carried out on commercial pig farm randomly distributing 60 animals (crossbred Large White × Swedish Landace × Pietrain) to four different boxes with full concrete floor and Big Dutchman® feeders. Animals (initial body weight 30.85 ± 0.30 kg) were fed over a period of 98 days with a standard feed mixture based on corn and soybean for fattening up to 60 kg (ST-1; Se concentration 0.058 mg/kg DM) and a mixture for fattening up to 100 kg (ST-2, Se concentration 0.050 mg/kg DM; Table 1). The groups were fed different dietary selenium treatments as follows: C-0.3 mg/kg DM organic Se, E1-0.5 mg/kg DM sodium selenite, E2-0.5 mg/kg DM organic selenium; E3-0.5 mg/kg DM organic Se+0.2% natural zeolite clinoptilolite (Zeotex; Mevex Ltd, Zagreb, Croatia; Table 2). The dietary treatment with 0.3 mg/kg DM organic Se in C was used as a standard recommended by NRC (22) (0.3 mg/kg for weaning pigs to 0.15 mg/kg for growing pigs). Because of the lack of Se in the soil, and consequently in plants, the current regulation allows up to 0.3 mg/kg of Se in the diet for all pigs, hence that level was used in our research as a gold standard. Feed and water were offered *ad libitum*, with an average ambient temperature of 25°C during the day and 16°C during the night. Pigs were weighted at the beginning of the trial, 50th and 98th day. The trial was conducted according to Directive 2010/63/EU of the European Parliament (2010) on the protection of animals used for scientific purposes, according to Croatian Animal Protection Act, other legal acts regarding the animal welfare and with Approval of Bioethics Committee for Research on Animals University in Osijek (2158-94-02-21-02).

**Table 1:** Ingredients and chemical composition of the experimental diets.

**Ingredient**	**ST - 1**	**ST - 2**
	**%**	**%**
Dry corn	30	30
Wet corn	–	20
Barley	33	26
Bran	8	5.5
Soybean meal	7	11
Extruded soybean	10	–
Sunflower meal	–	5
Sunflower cake	9	–
Calcium carbonate	1.5	1
Monocalcium phosphate	1	1
Mineral premix^*^	0.5	0.5
Total	100	100
Chemical composition		
Dry matter	87.50	87.50
Crude protein	16.10	13.90
Fat	3.70	3.30
Crude fiber	5.30	6.90
Ash	4.93	5.84
Na	0.14	0.13
Ca	0.63	0.66
P	0.45	0.40
Se	0.0588 mg/kg DM	0.0507 mg/kg DM

* *Mineral premix: Iron 16,000 mg, Copper 4,000 mg, Manganese 8,000 mg, Zink 16,000 mg, Iodine 150 mg, Cobalt 40 mg, vitamin A 1,000,000 IU, vitamin D3 100,000 IU, vitamin E 2,400 mg, vitamin K3 400 mg, vitamin B1 400 mg, vitamin B2 600 mg, vitamin B6 400 mg, vitamin B12 3 mg, Nicotine acid 3,000 mg, Pantothenic acid 2,000 mg, Colin chloride 100,000 mg*.

**Table 2:** Selenium supplementation and experimental design.

**Group**	***N***	**Supplementation**
Control	20	0.3 mg organic selenium
E1	20	0.5 mg inorganic selenium
E2	20	0.5 mg organic selenium
E3	20	0.5 mg organic selenium + 0.2% clinoptilolite^*^

* *Zeolite clinoptilolite composition: SiO_2_ 63–68%, Al_2_O_3_ 11–14%, Fe_2_O_3_ 0.8–2.5%, MnO 0.01–0.3%, CaO 2.5–4.5%, MgO 0.8–1.5%, Na_2_O 0.8–1.5% K_2_O 1–2% L.I. 10.5–14.5%*.

### Chemical Composition of the Diets

The feed composition was determined using standard methods (AOAC, 2006). Feed samples were analyzed for dry matter (24 h at 103°C; ISO 6496:2001) and ash by ISO 5984:2002. The crude protein (CP) content was estimated from the nitrogen concentration according to the Kjeldahl method using Kjeldahl steam distillation for nitrogen (Behr, Stuttgart, Germany; ISO 5983-2:2005). The Universal Extraction System B-811 (Buchi, Flawil, Switzerland) was used to analyze ether extract (EE; ISO 6492:1999). The ingredients and chemical compositions of the experimental diets are shown in Table 1. Selenium supplementation was added according to the experimental model, as shown in Table 2.

### Sample Collection

Blood samples were collected from *v. cava cranialis* in vacuum tubes with heparin as an anticoagulant (Beckton Dickinson, Plymouth, UK) for selenium concentration and antioxidative enzymes. Other samples were taken with the EDTA for flow cytometry at 10 a.m. at each sampling point on eight pigs from each group. To measure selenium concentration in blood, immune parameters, and enzymes in antioxidant status blood samples were collected at the beginning of the trial (day 0) and at the 50th, 71st, and 98th days of trial. The samples of the *m. longissimus dorsi* and liver were taken at the end of the trial, subsequent to slaughter, at the abattoir, for the determination of selenium concentration. A piece of ~7 cm from the left *longissimus dorsi* muscles were excised, starting at the joint between 12th and 13th thoracic vertebrae. A liver sample was taken from the apex of the left medial lobe (*lobus hepatis sinister medialis*).

### Determination of Selenium Concentration in Blood, Muscle, and Liver Tissue by ICP-OES

The concentration of selenium in blood, muscle, and liver tissue was determined using inductively coupled plasma optical emission spectrometry (ICP-OES, PerkinElmer Optima 2100 DV, USA). For the pre-reduction of Se, 20 mL of samples were placed in a clean 50 mL beaker, and 20 mL of concentrated HCl was added to reduce Se^6+^ to Se^4+^ (12). The mixture was heated to 90°C and cooled to room temperature. The wavelength for Se determination was 196.026 nm. For the quality control of the analytical method, the certified reference material, Cabbage (NCS ZC 73012, China National Analysis Center) was digested, and the total concentration was determined for method validation. The recovery rates of Se were within the range of 10%. All samples were analyzed in triplicate.

### Determination of Circulating Immune Cell Subsets by Flow Cytometry

The monoclonal antibodies (mAbs) reactive with swine leukocyte surface molecules (i.e., cluster of differentiation [CD] antigens) that we used for the identification and quantification of patterns of the lymphoid cell subsets are listed in Table 3. Anti-swine mAbs to CD45^+^ (clone K252-1E4), CD4^+^ (clone 74-12-4), CD8^+^ (clone 76-2-11), and CD21^+^ molecules (clone BB6-11C9.6) were obtained from Abcam (Cambridge, UK). The cell suspensions were prepared and incubated with mAbs (50 μl/10^6^ cells) in single color flow cytometry to determine the percentage of positively stained cells (23). Flow cytometric analysis of the positively stained cells expressing CD45^+^, CD4^+^, CD8^+^, or CD21^+^ molecules were performed for each animal, and the data are presented as the arithmetic mean ± pooled standard error of mean (mean ± SEM). The fluorescence of the mAb-labeled porcine lymphoid cells was quantified using a Coulter EPICS-XL flow cytometer (Beckman Coulter Miami FL, USA) as reported earlier (24). The isotype-matched mouse immunoglobulins were used to detect non-specific fluorescence in control cell suspensions.

**Table 3:** Murine monoclonal antibodies specific to swine leukocyte surface CD (cluster of differentiation) antigens and conjugates used in cytometric immunophenotyping of peripheral blood lymphoid cells from growing pigs fed with different Se sources and concentrations.

**Clone (mAb)/pAb**	**Isotype**	**mAb specificity**	**Conjugate**	**Targeted cells/molecule**
74-12-4	IgG2b	CD4^+^	Pe/Cy5®	Helper T lymphocytes
76-2-11	IgG2a	CD8^+^	Phycoerythrrine	Cytolytic T lymphocytes
K252-1E4	IgG1	CD45^+^	FITC	Lymphoid cells
BB6-11C9.6	IgG1	CD21^+^	FITC	B lymphocytes

### Determination of Antioxidative Enzymes: GSH-Px and Glutathione Reductase (GR)

For antioxidative enzyme determination, blood was centrifuged for 5 min at 1,500 g. After removing the plasma and buffy coat, erythrocytes were washed three times by resuspension in 0.9% NaCl and centrifuged for 5 min at 2,000 rpm after each wash. Erythrocyte samples were frozen at −80°C until analysis. For the determination of antioxidant enzyme activity in erythrocyte lysate, packed erythrocytes were hemolyzed for 10 min at +4°C by a 4-fold dilution with ice-cold Milli Q water, and the lysate was clarified by centrifugation (10, 000 g, 5 min, +4°C) for analysis. Activity of enzymes (GSH-Px and GR) were determined by spectrophotometry in erythrocytes using the Ransel® and Glutathione Reductase® assay kits (Randox Laboratories Ltd, London, UK).

### Statistical Analysis

For the evaluation of the treatment on the variability of body weight, immune parameters, oxidative parameters, and selenium concentration in blood following statistical model was used:

$$\begin{array}{l} y_{\text{ijk}}=\mu +T_{\text{i}}+D_{\text{j}}+T_{\text{i}}xD_{\text{j}}+e_{\text{ijk}} \end{array}$$

Where:

y_ijk_ = estimated trait (body weight, immune parameters, oxidative parameters and selenium concentration in blood);
μ = intercept;
T_i_ = fixed effect of treatment i (groups = C, E1, E2, and E3);
D_j_ = fixed effect of measurement day j (j = 0, 50, 71, and 98 day of measurement);
e_ijk_ = residual.

For the evaluation of the treatment on the variability of selenium concentration in tissues (muscle and liver) following statistical model was used:

$$\begin{array}{l} y_{\text{ijk}}=\mu +T_{\text{i}}+e_{\text{ijk}} \end{array}$$

Where:

y_ijk_ = estimated trait (selenium concentration in tissues);
μ = intercept;
T_i_ = fixed effect of treatment i (groups = C, E1, E2, and E3);
e_ijk_ = residual.

The significance of the differences between the analyzed traits due to fixed effect of treatment was tested by Fischer's test at level of *P* < 0.05; PROC GLM procedure in STATISTICA using TIBCO Software Inc., 2018.

## Results

### Selenium Concentration in Blood, Muscle, and Liver

**Figure 1:**
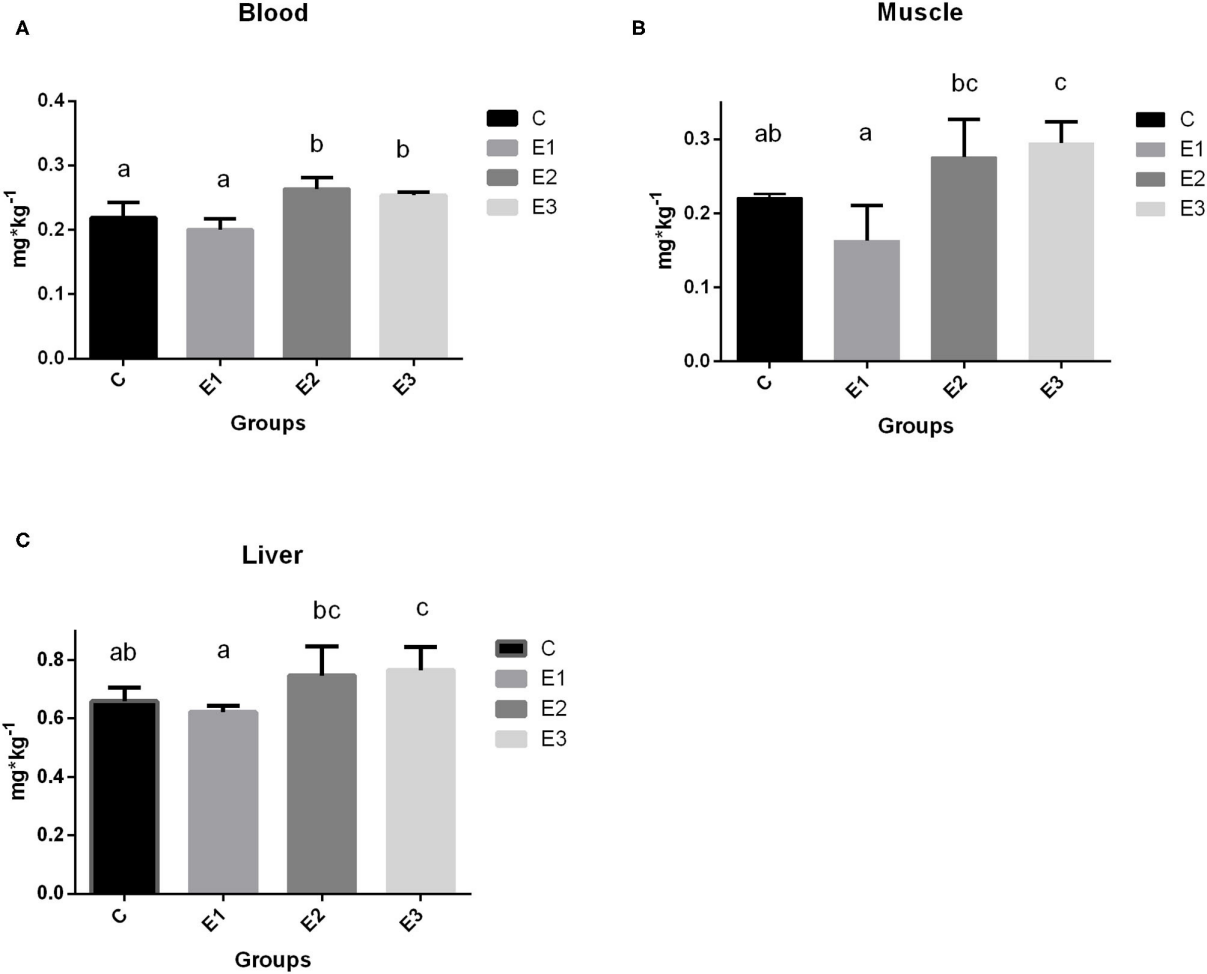
Selenium concentration in blood **(A)**, muscle **(B)**, and liver **(C)** tissue of growing pigs fed with different sources and concentrations of selenium; C-0.3 mg/kg DM organic Se, E1-0.5 mg/kg DM inorganic Se, E2-0.5 mg/kg DM organic Se; E3-0.5 mg/kg DM organic Se+ 0.2% zeolite clinoptilolite. Values are means ± SEM. Bars with different superscript letters (a, b, c) were different, *P* < 0.05.

The growing pigs had similar body weights throughout the experimental period (Table 4). Se concentration in blood was higher (*P* < 0.05) in the E2 and E3 groups than in the C and E1 groups, and clearly depended on the Se source and quantity (Figure 1A). Higher (*P* < 0.05) Se concentrations were determined in the muscle and liver in the E2 and E3 groups and compared with those of the E1 group, and the concentrations of the E3 group were compared with those of the C group (Figure 1). The Se concentrations after dietary addition in the E1 group were lower (*P* < 0.05) in blood than in the E2 and E3 groups fed with the same amount of the organic source of Se (Figure 1). Pigs fed with a lower amount of organic Se (C) had a numerically higher concentration of Se compared with the E1 group (Figure 1).

**Table 4:** Average body weight (kg) of growing pigs fed different Se source and concentration with *P*-values of effects tested in statistical model.

	**Group**					***P*** **-value**		
**Day**	**C**	**E1**	**E2**	**E3**	**SEM**	**Group**	**Day**	**Group × Day**
0	31.75	31.13	30.67	29.85	0.30	0.4963	0.0001	0.5136
50^*^	63.96	62.48	60.05	62.77	0.66			
98^**^	98.47	97.53	98.73	98.41	0.80			

* *End of first fattening stage (ST-1 feed)*;

** *End of second fattening stage (ST-2 feed)*.

*C-0.3 mg/kg DM organic Se, E1-0.5 mg/kg DM inorganic Se, E2-0.5 mg/kg DM organic Se; E3-0.5 mg/kg DM organic Se+0.2% zeolite clinoptilolite*.

### Recruitment of Immune Cell Subsets

**Figure 2:**
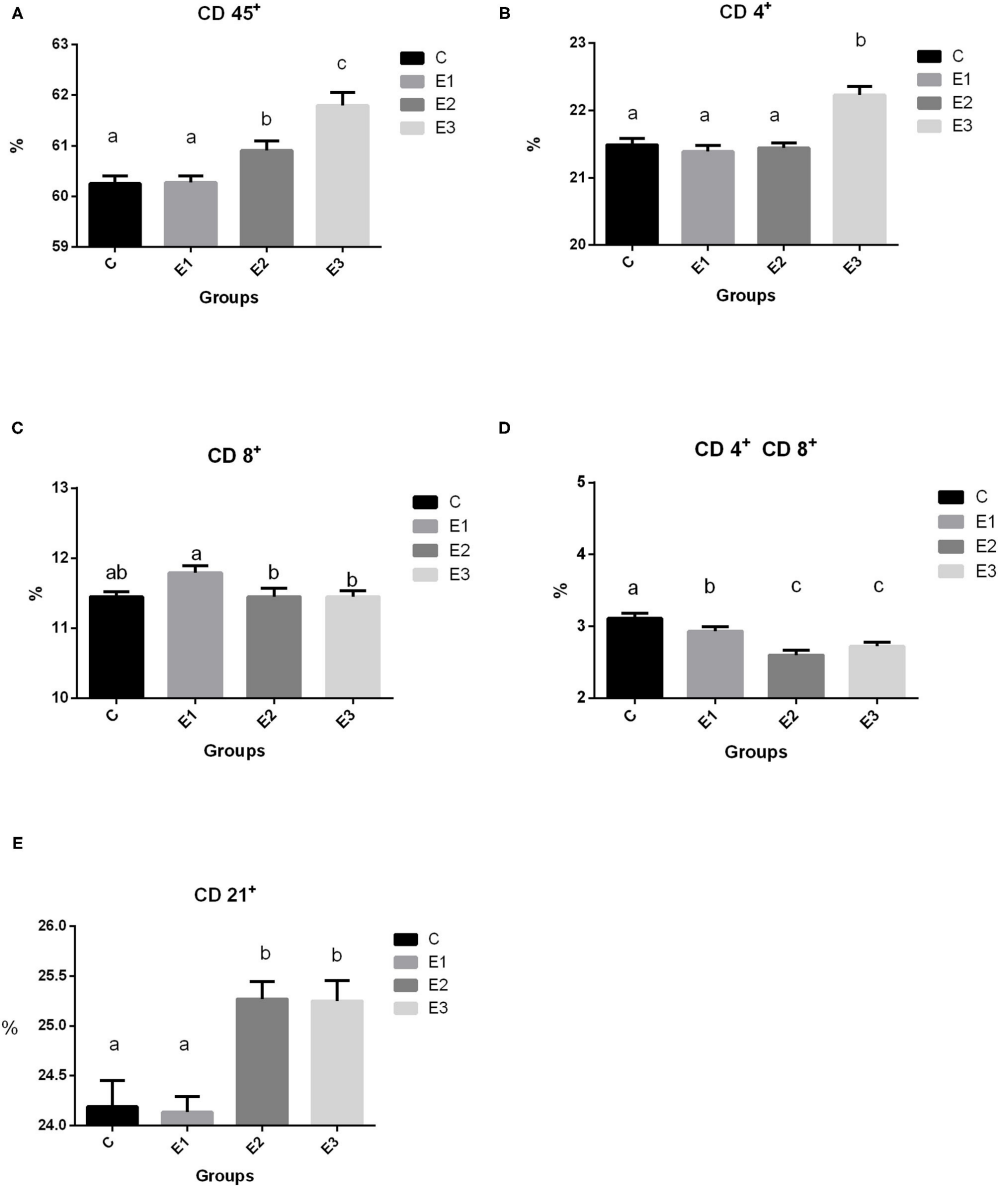
Proportion of lymphoid cell subsets (**A** CD 45+, **B** 4+, **C** CD 8+, **D** CD 4+ CD8+, **E** CD 21+) in the peripheral blood of growing pigs fed with different sources and concentration of selenium. C-0.3 mg/kg DM organic Se, E1-0.5 mg/kg DM inorganic Se, E2-0.5 mg/kg DM organic Se; E3-0.5 mg/kg DM organic Se+0.2% zeolite clinoptilolite. Values are means ± SEM. Bars with different superscript letters (a, b, c) were different, *P* < 0.05.

Recruitment of circulating immune cell subsets assessed by the cytometry analysis of proportions of CD45^+^ lymphoid cells, CD4^+^, CD8^+^, and CD 4^+^CD8^+^ T cells, as well as of CD21^+^ B cells in the peripheral blood of growing pigs fed with different Se sources for 98 days are shown in Figure 2. A higher (*P* < 0.05) proportion of total leucocytes was noted in the pigs of the E2 and E3 groups compared with groups C and E1. Interestingly, a higher (*P* < 0.05) proportion of CD4^+^ lymphocytes was observed in the pigs from the E3 group, while the proportion of CD8^+^ lymphocytes was higher (*P* < 0.05) in the E1 group. Pigs from the E2 and E3 groups had lower (*P* < 0.05) double-positive CD4^+^CD8^+^ lymphocytes than pigs from the C and E1 groups. A higher (*P* < 0.05) proportion of CD21^+^ lymphocytes was measured in the E2 and E3 groups compared with the C and E1 groups.

### Antioxidant Enzymes Activity

The highest (*P* < 0.01) activity of GSH-Px in pig erythrocytes in the E3 group was compared with that in the other groups. Dietary addition of higher concentrations of organic and inorganic Se increased the activity of GR in relation to the C group, with lower Se content in the feed.

## Discussion

Selenium is a crucial trace element for antioxidant and immune functions in animals and humans (25). Many studies have confirmed that organic Se acts as a real antioxidant, unlike inorganic Se, which can act as a prooxidant. Bearing in mind that many studies have proved that Se-enriched yeast is superior to inorganic sources in resorption and deposition (26–28), we used a source that contained organic Se at a concentration of 0.3 mg/kg DM in a control group. The experimental groups contained higher levels of Se (0.5 mg/kg) in inorganic (E1) and organic (E2) forms, and higher levels of organic Se combined with natural zeolite clinoptilolite (E3).

Selenium deposition in muscle tissue is a good indicator of the Se status of animals (17). Organic Se has an important benefit compared with inorganic Se due to the fact that selenomethionine is utilized by the tissues as an amino acid. The Se reserve in tissues can be mobilized for subsequent selenoprotein synthesis. Selenomethionine from Se yeast is known to be the best source for increasing the level of Se and depositing Se in tissues (29). In our study, higher (0.5 mg/kg) dietary addition of Se-enriched yeast increased Se concentration in muscle and liver as compared to the addition of inorganic Se at 0.5 mg/kg (E2, E3 vs. E1). The addition of Se combined with zeolite clinoptilolite (E3) resulted in higher concentrations of Se in the muscle and liver compared with the C group. Similarly, Jiang et al. (30) found the highest deposition of Se in meat after the addition of 0.3 mg/kg Se with 1.5 mg linseed oil. The extent of tissue deposition in liver and muscle in our study was comparable to the finding of Zhan et al. (31). Moreover, there was no difference between Se concentration in blood, muscle, and liver between basal diet according to the NRC (22) (C; 0.3 mg/kg DM) and inorganic Se supplementation (E1; 0.5 mg/kg DM), which is in agreement with the results of Mohamed et al. (32) in broiler chicken. The reason for an even higher Se concentration in the groups with zeolite addition (E3) compared with E2 is due to a positive effect of a zeolite as an adjuvant (18). Clinoptilolite is the most prevalent zeolite in the nature. Its therapeutic applications are numerous; it is used for the maintenance of body's pH value, reduction of free radicals, neutralization or elimination of toxins and heavy metals, the improvement of tissue oxygenations, etc. (33). Clinoptilolite is a great source of silicon in the form of orthosilicic acid which protects the body from heavy metals (34). The zeolites have a protective effect in intoxication. That effect is evidenced by researchers who observed that the clinoptilolite could have some protective effect in organophosphorus poisoning in sheep, in lead intoxication in mice, and in pigs which have received CdCl2 (35). The toxic elements generate reactive oxygen (ROS) or nitrogen (RNS) species that damage lipids, proteins, and deoxyribonucleic acid (DNA). Selenium forms a complex with transition metals and seleno-compounds that decrease their toxicity. Both complex formation and oxidative damages contribute to a decrease of seleno-compound concentrations like Se-methionine (36). A zeolite supplementation reduces the toxic elements concentrations by combining with them in gastrointestinal tract. In that way less complex formation and oxidative damages allows for a more Se-methionine to be deposited in tissues. The other possible way of clinoptilolite action is explained through a better Se absorption, based on an observation in Wistar rats which have received the zeolite for 34 days. Some modifications of intestinal microvilli were observed. The microvilli length was higher and the number of microvilli per square μm was higher (37), which could point to a better absorption capability. The other experiment with the newborn calves has demonstrated a better passive transfer of immunoglobulin into newborns (38).

In human medicine there are studies that support the beneficial properties of purified natural clinoptilolite as an anti-diarrheic treatment (39). More recent studies performed on aerobically trained subjects, highlighted the positive effects of zeolites on intestinal wall integrity (40).

To summarize all above zeolite decreases heavy metal and mycotoxin absorption from intestines and reduces oxidative burden and at the same time improves capacity for selenium absorption which at the end result in better selenium bioavailability and higher selenium concentrations in tissues.

In our study, we found higher (*P* < 0.05) leukocyte common antigen (CD 45^+^) in the E2 and E3 groups than in the C and E1 groups, which means that Se and Se+Zeolite increase cellular immunity. Regarding the lymphocyte markers, there was a higher (*P* < 0.05) proportion of the CD4^+^ markers in the E3 group, which could be a positive effect attributed to the addition of Se+zeolite, because the E2 group with the same Se addition did not differ from the control group (C). Ivory et al. (41) found that Se supplementation did not enhance T-cell proliferation. In this case, the response was boosted by flu vaccination. In our case, the effect of increased CD4^+^ cell subsets were due to the non-specific stimulation by zeolite Se+zeolite addition (Figure 2B). In contrast, higher Se supplementation increased CD4^+^ proliferation in mice at low and medium doses (42) by modulating free thiol levels and specific signaling events during CD4^+^ activation. We can only speculate which dose is optimal for eliciting immune and antioxidative effects in the live organism and why in our research CD4^+^ was only increased in the E3 group. Namely, the proliferation of T cells is related to oxidized and reduced glutathione, which is a very important molecule for cell protection against oxidative damage. As reported by Ivory et al. (41) the addition of onion (rich in flavonoids) with a low Se dose increased the granzyme and perforin content of CD8^+^ cells, while the opposite effect was observed at higher Se doses. The pigs fed with a higher concentration of organic Se in the experimental groups had a lower (*P* < 0.05) ratio of CD8^+^ lymphocytes when compared to the pigs fed with the same concentration of inorganic Se. This is in accordance with Taylor et al. (43) who found that a higher Se content induces a plentiful production of selenoprotein P, which overlaps with the genes encoding several T cell-associated genes. Products from the cytolytic granules from CD8^+^ cells are also influenced by the dosage and form of Se; lower granzyme content with higher Se dosage has been reported by Ivory et al. (41) While Broom et al. (44) found a significant increase in CD4^+^ and CD8^+^ levels and a numerical increase in B lymphocytes, which could be explained by the lower Se status in the patients and the immunity elicited by the poliovirus vaccine.

Dual-positive T lymphocytes in the peripheral circulation have been identified by researchers as part of a specific immune response, including a broad spectrum of T lymphocytes involved in the antiviral immune response in pigs (45, 46). A smaller proportion of them was found in the peripheral circulation, but it was also proven that T lymphocytes increased by 20% during viral infections. This cell population comprises mature effective lymphocytes specific to the repertoire of antigens encountered in the past and latent and highly persistent viral infections (47). Our results showed a lower proportion of dual positive T lymphocytes with the addition of organic Se and in combination with zeolite, which is probably due to a lack of proper challenge. Interestingly, higher dietary addition of Se elicited a share of the CD21^+^ lymphocytes, but only in the E2 and E3 groups, which is related to the signal transducer and set of cytokines that regulate this path of immunity. Confirmation of this could be found in Hofmann and Berry (48), who noticed that Se affects different types of immune responses in different ways, depending on the starting Se status. Avery and Hoffmann (49) concluded that the use of selenium to increase innate immunity may be enhanced when prescribed along with other nutritional antioxidants (which is zeolite in our case). Adaptive immunity is affected by selenium intake, including the activation and function of T and B cells.

**Figure 3:**
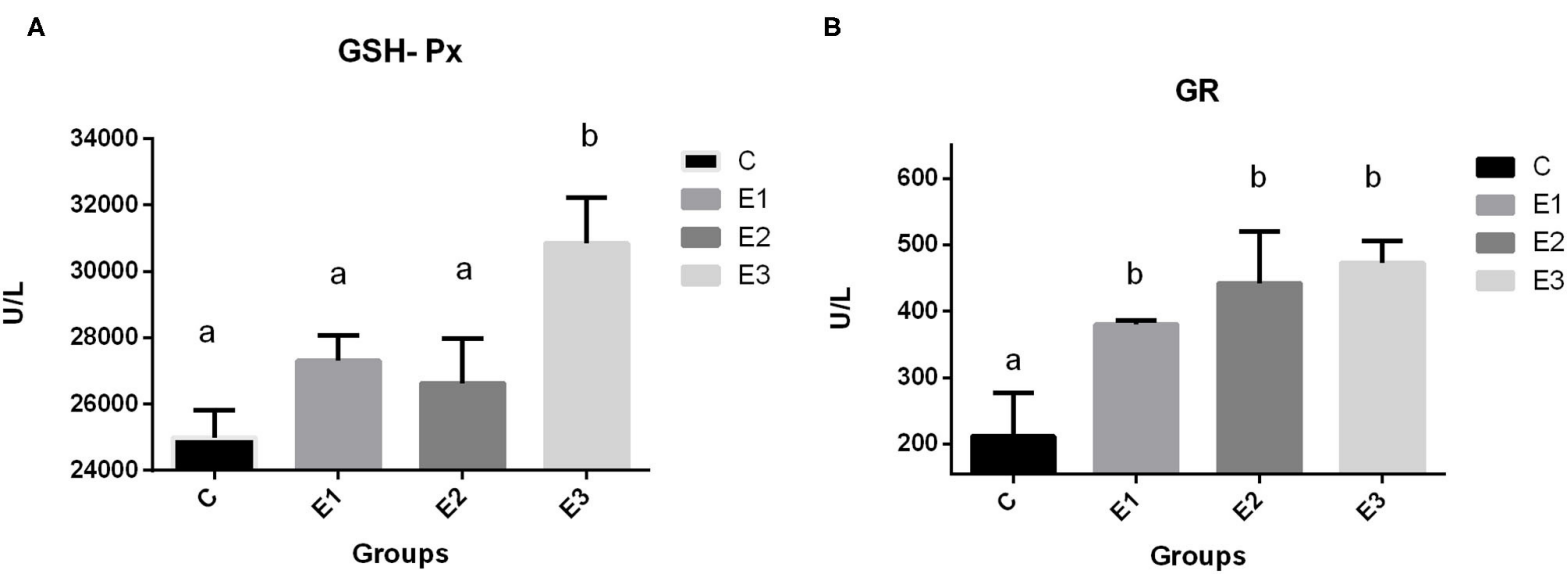
Activities of glutathione peroxidase (**A** GSH-Px) and glutathione reductase (**B** GR) in blood of growing pigs fed with different source and concentrations of selenium. C-0.3 mg/kg DM organic Se; E1-0.5 mg/kg DM inorganic Se; E2-0.5 mg/kg DM organic Se; E3-0.5 mg/kg DM organic Se+ 0.2% zeolite clinoptilolite. Values are means ± SEM. Bars with unlike superscript letters (a, b, c) were different, *P* < 0.05.

Because Se is included in many selenoproteins in the body and the most commonly researched is GSH-Px, which is involved in protecting cells from oxidative damage, dietary deficiency of Se causes redistribution of intracellular Se among the selenoproteins and GSH-Px protein (50). The high dependency of GSH-Px activity on Se source and concentration in the diet has been confirmed previously by many authors (51, 52). A meta-analysis by Bermingham et al. (53) found a significant correlation of GSH-Px activity with Se dose and form, which is consistent with the results of Mahan et al. (54). In contrast, Oliveira et al. (16) reported a linear reduction in plasma GSH-Px activity with increased supplementation levels of organic Se, and a linear increase in hepatic GSH-Px activity with increased supplementation, regardless of the type source in post-weaning piglets. In our research, the higher concentrations of dietary organic selenium and zeolite combination increased (*P* < 0.05) GSH-Px activity in E3 group (Figure 3) when compared to the other groups. A higher GSH-GPx activity in this group is explained through a higher Se-methionine availability due to a lesser Se complex formation and lesser toxic elements ROS and RNS damage, as previously described by a higher Se tissue concentrations. An increase in dietary Se concentration, either organic or inorganic, increased GR activity in blood.

Glutathione reductase (GR) is an oxidoreductive enzyme primarily responsible for the maintenance of intracellular glutathione concentration, playing an important role in the protection of cellular components with regard to an oxidative damage, especially that of erythrocytes. It has been demonstrated that, under the conditions of an oxidative stress, the activities of the GR antioxidant enzyme have increased in serum or in erythrocytes, but, in our case, an increase in the GR activity is in connection with GSH-Px one. If selenium is sufficiently consumed in diet, the GSH-Px enzyme activity increases, and when the substrate increases, the enzyme levels increase, too, to bind the substrate, reaching the maximal steady-state rates. If the GSH-Px activity is sufficient, glutathione disulfide is produced at the levels sufficient to stimulate the GR and prevent its deactivation by the NADPH (55). Under these investigation conditions, a higher GR activity in the experimental groups signify that more glutathione was restored. Therefore, a better antioxidative defense was achieved.

Our research circumstantiates the benefits of feeding the growing pigs with slightly over the NRC recommended levels of organic selenium (0.5 mg/kg DM) with the zeolite on selenium concentrations in tissues for human consumption, additionally it improves immune defense and andioxidative capacity of the growing pigs. A future research should investigate a potential of different zeolite concentration in combination with selenium on selenium tissue deposition as a benefit for humans and immune and oxidative defense as a benefit for animals.

## Conclusions

The addition of organic Se is favorable to the inorganic Se in the same concentration (0.5 mg/kg DM). Dietary addition of 0.5 mg/kg DM organic Se with a zeolite addition (0.2%) increased Se deposition in liver, muscle, and blood, compared to lower organic Se concentration (0.3 mg/kg) which has a positive implication for the pig industry as a way of producing a functional food for human benefit. A significant increase in the immune and antioxidative parameters in the group of pigs fed the combination of selenium and zeolite had the positive effect on animal health.

## Data Availability Statement

The raw data supporting the conclusions of this article will be made available by the authors, without undue reservation.

## Ethics Statement

The animal study was reviewed and approved by Bioethics Committee for Research on Animals J. J. Strossmayer University of Osijek (2158-94-02-21-02).

## Author Contributions

TŠ and MŠ designed this study, planned and carried out the experiments and measurements, and drafted the manuscript. MÐ carried out the measurements and analysis and helped to write the manuscript. ZL, VP, and MP helped with the analysis of the samples. VG has made statistical analyses. All the authors read and approved the final manuscript.

## Conflict of Interest

The authors declare that the research was conducted in the absence of any commercial or financial relationships that could be construed as a potential conflict of interest.

## Publisher's Note

All claims expressed in this article are solely those of the authors and do not necessarily represent those of their affiliated organizations, or those of the publisher, the editors and the reviewers. Any product that may be evaluated in this article, or claim that may be made by its manufacturer, is not guaranteed or endorsed by the publisher.
